# Cold Atmospheric Plasma Targeting Hematological Malignancies: Potentials and Problems of Clinical Translation

**DOI:** 10.3390/antiox11081592

**Published:** 2022-08-17

**Authors:** Sebastiano Gangemi, Claudia Petrarca, Alessandro Tonacci, Mario Di Gioacchino, Caterina Musolino, Alessandro Allegra

**Affiliations:** 1 Unit of Allergy and Clinical Immunology, Department of Clinical and Experimental Medicine, School of Allergy and Clinical Immunology, University of Messina, 98125 Messina, Italy; 2 Department of Medicine and Aging Sciences, G. D’Annunzio University, 66100 Chieti, Italy; 3 Center for Advanced Studies and Technology, G. D’Annunzio University, 66100 Chieti, Italy; 4 Clinical Physiology Institute, National Research Council of Italy (IFC-CNR), 56124 Pisa, Italy; 5 Institute for Clinical Immunotherapy and Advanced Biological Treatments, 65100 Pescara, Italy; 6 Division of Hematology, Department of Human Pathology in Adulthood and Childhood “Gaetano Barresi”, University of Messina, 98125 Messina, Italy

**Keywords:** cold atmospheric plasma, hematological malignancy, oxidative stress, apoptosis, epigenetics, angiogenesis, acute myeloid leukemia, chronic myeloid leukemia, multiple myeloma

## Abstract

Cold atmospheric plasma is an ionized gas produced near room temperature; it generates reactive oxygen species and nitrogen species and induces physical changes, including ultraviolet, radiation, thermal, and electromagnetic effects. Several studies showed that cold atmospheric plasma could effectively provoke death in a huge amount of cell types, including neoplastic cells, via the induction of apoptosis, necrosis, and autophagy. This technique seems able to destroy tumor cells by disturbing their more susceptible redox equilibrium with respect to normal cells, but it is also able to cause immunogenic cell death by enhancing the immune response, to decrease angiogenesis, and to provoke genetic and epigenetics mutations. Solutions activated by cold gas plasma represent a new modality for treatment of less easily reached tumors, or hematological malignancies. Our review reports on accepted knowledge of cold atmospheric plasma’s effect on hematological malignancies, such as acute and chronic myeloid leukemia and multiple myeloma. Although relevant progress was made toward understanding the underlying mechanisms concerning the efficacy of cold atmospheric plasma in hematological tumors, there is a need to determine both guidelines and safety limits that guarantee an absence of long-term side effects.

## 1. Introduction

### General Consideration Con Cold Atmospheric Plasma

In physical science, plasma is defined as the fourth state of matter, which is usually generated at high temperature or at low pressure. It is frequently characterized as an ionized gas created by the fragmentation of polyatomic gas particles or the subtraction of electrons from monatomic gas shells [1]. Nevertheless, not all ionized gases that enclose charged molecules can be considered plasma [2,3]. In fact, plasma must feature macromolecular neutrality, and in the lack of exterior influences, the resulting electric charge is null. Furthermore, plasma must have Debye shielding, where the charged molecules are organized to successfully protect electrostatic fields within the space of a Debye length (the ratio of the electron thermal velocity divided by the plasma frequency).

Consequently, plasma can be described as a quasi-neutral gas that includes ionized atoms and several interrelating free electrons that present similar behaviors provoked by long-distance Coulomb forces. Finally, the charged-molecule activity in plasma induces the formation of electric fields and produces the onset of magnetic fields [4].

Cold atmospheric plasma (CAP) is a plasma in which heavyweight molecules have temperatures that are near to room temperature due to weak elastic collisions throughout the discharge procedure [5]. CAP can be produced at an atmosphere milieu, which makes it useful for applications in the biological field. In fact, CAP produces several consequences, such as electromagnetic (EM), thermal, and UV effects [6,7].

**Figure 1:**
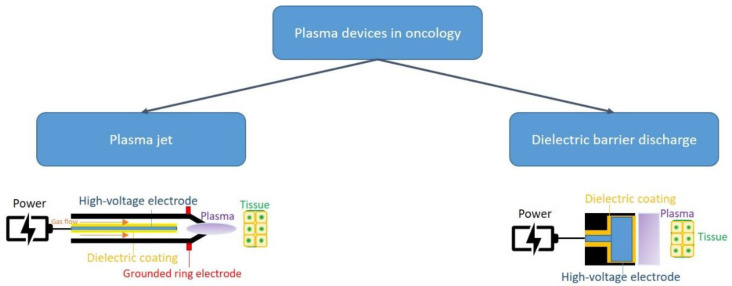
Common plasma devices in oncology and their functioning.

Different methodologies have been employed to produce CAP, including piezoelectric direct discharge technology and pulsed atmospheric arc technology, both of which have diverse pros and cons, as every technique is able to generate a diverse pattern of results. However, the production of CAP offers biological elasticity and the possibility of influencing different cellular events. Characteristic conformations include a plasma jet and dielectric barrier discharge [8] (Figure 1).

As for possible CAP medical applications, important technical improvements have allowed enhancements of the efficiency of CAP apparatuses and their aptness for medical application. For instance, it was possible to increase diffusion deepness to 5 cm and to prevent problems due to high voltage, gas liberation, and intra-organic discharge generation [9,10].

The main elements of the biological effects of CAP include reactive oxygen species (ROS) and nitrogen species (RONS), electrons, ions, and UV photons [11]. When CAP is produced, it includes reactive species, such as hydroxyl groups, hydrogen peroxide, ozone, and nitrogen oxides. Next, the reactive species interact with the extracellular compartment to generate additional reactive species, including oxonium ions, nitrogen dioxide, nitrogen oxyanions, peroxynitrite, and hydroperoxyl radicals. The components and amounts of these molecules can be regulated and predetermined for different purposes, in particular medical ones.

In the clinical setting, the CAP devices employed are of three different types: devices based on direct discharge, devices relying on indirect discharge, and hybrid forms. Direct dielectric barrier discharge (DBD) occurs between an elevated voltage electrode and a grounded one. After an early gas break, ions and electrons in these apparatuses are blocked by the dielectric barrier. The electric field of electrons and ions then protects the electric field from the external source [12,13,14].

The most important DBD devices include plasma jet and surface plasma, but the extent of their actions is different. The surface plasma source produces constant plasma on the plane of the dielectric plate, while the plasma jet source produces plasma in the discharge area, in which the action range is small. Several experiments have reported a biological effect of these two plasma sources, but the results produced by the various plasma sources on living cells may be different [15].

Indirect discharge is produced by devices that are analogous to direct discharge devices, but the carrier gas guides the plasma discharge. Thus, the discharge does not impact the structure between the two electrodes, but advances in the gas stream course. Although this process allows the target to be placed outside the device, smaller quantities of ROS and RONS species are generated and the discharge is more difficult to regulate than it is with DBD tools. A further difference is that plasma produced by indirect devices has greater UV effects but does not generate electric current. The hybrid plasma tools merge these characteristics, but they are presently employed only at the experimental level.

The plasma-activated medium (PAM) is an effective method for affecting cells that are deeply placed within the tissues. Reactive species can be subcutaneously administered into tissues in a liquid shape or produced in situ via micron-sized fonts, and CAP can penetrate up to 5 cm into tissues [16]. The skin is strong in stopping CAP-originated reactive species and allows only partial diffusion of RONS that are mostly blocked by the stratum corneum layers [17]. Recently, a slight invasive in situ plasma source, invivoPen, was developed, with advantages over PAM in enhancing immune response and decreasing inflammation. The latest innovations on plasma-induced immune cell death [18] essentially modify the possibility of reaching deep tissues by CAP, as plasma-caused effects are quickly transferred by immune cells through the body and into distant cells.

Beyond the chemical species transferred to cells from CAP, the contacts between physical effects due to plasma and the molecules at the gas-liquid edge, and within the liquid, can produce several effects, such as the generation of photons, electric fields, and shockwaves [19,20]. These elements could interrelate with the cell surface to obtain many, specific cell effects [21,22].

Furthermore, an important decrease in cell proliferation by plasma-stimulated cell culture medium and plasma-stimulated Ringer’s lactate solution in in vivo experiments was reported. Several reports stated that the antiproliferative action of plasma-activated liquids can be due to RONS effects [23].

CAP demonstrated encouraging possibilities in several fields, such as wound healing, tissue ablation, disinfection, and tumor therapy [24,25,26,27,28,29,30,31]. In fact, oxidative stress and RONS could directly modify the micro-environment of tumor cells and change their behavior [32,33,34,35,36,37].

## 2. CAP and Cancer

Plasma can successfully provoke cell death in several varieties of tumor cells, including glioma, cervical cancer, and colon cancer cells [36,37,38,39,40]. On the other hand, CAP does not appear to alter normal cells [41].

**Figure 2:**
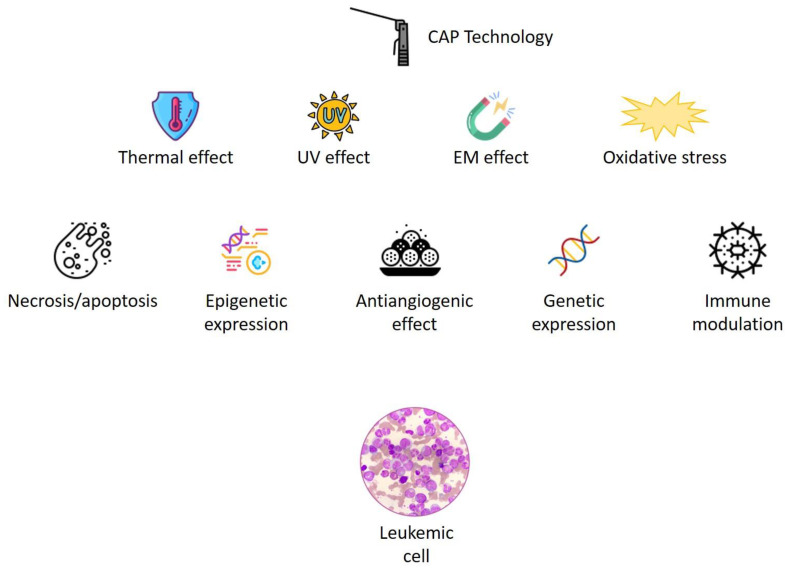
Effects and mechanisms of CAP on leukemic cells.

Several mechanisms seem to be able to justify the actions exerted by CAP on cancer cells (Figure 2). Among other effects, the effects produced on gene and epigenetic expression, apoptotic and angiogenetic dynamics, and the modulation of the immune system seem to be particularly relevant. Essential pathways, such as ERK, TGF-beta, DNA repair, PI3K/Akt signaling, and HIF, which are fundamental in stimulating tumors [14,15], are also controlled by CAP [42,43,44].

### 2.1. CAP and Cell Death

The CAP-stimulated programmed cell death is due to well-known apoptotic pathways. The main CAP-stimulated tumor cells’ death is that of the caspase-dependent apoptosis mechanism, although a minority of studies stated the possibility of a caspase-independent death in the CAP-exposed tumor cells [45,46].

In CAP-treated tumor cells, several phenomena have been reported, such as the delivery of Cyt c into cytosol, the reduction of mitochondrial transmembrane potential, the DNA fragmentation, the production of p-p53/p73/p38/c-Jun N-terminal kinases, and caspases [47]. The activation of p53 is essential to the stimulation of apoptosis, but an apoptosis-independent cell death was described for the CAP-treated p53-mutated cancer cells. CAP provoked quick cell death by the amassing of lysosomes [48].

CAP is also able to change the dynamics of other modes of cell death, such as autophagy and cell necrosis. In fact, a recent experiment reported autophagy in the CAP-treated cancer cells. The increase of oxidative stress after CAP treatment has been considered as the main cause in stimulating autophagy in various cancer cell lines [49,50].

Finally, necrosis has also been reported in CAP-treated cancer cells. Employing a new experimental model, it was shown that melanoma cells were efficiently destroyed by the EM effect from a CAP jet source [51,52].

### 2.2. CAP and Angiogenesis

In addition to the stimulation of cell death, CAP is capable of reducing neoplastic progress via the inhibition of cancer angiogenic change. Several studies indicated that angiogenesis plays an important role in the pathogenesis of hematological malignancies [53,54]. The capability of CAP to reduce vascular endothelial growth factor expression in cancer cells was demonstrated in a study that evaluated growth factor, cytokines, and chemokines before and after CAP treatment. These findings indicated a suppressive effect of CAP on the angiogenic switch in cancer and its ability to modify the tumor milieu [55]. On the other hand, CAP could stimulate angiogenesis-related components in healthy cells, such as endothelial cells, fibroblasts, and skin keratinocytes, and this process could be useful in improving wound angiogenesis [56].

### 2.3. CAP and Epigenetic Changes

Although epigenetic modifications do not directly alter DNA sequence, they change gene expression [57,58]. These alterations are classified into those that influence the methylation amounts of nucleic acids and those that affect histone proteins. The contact period can establish the conclusive action of CAP on cell signaling pathways, leading to variations in the epigenetic status.

As for the relationships between CAP and DNA methylation, an experiment showed that the genes that reduced after 1 min exposure were essentially correlated to cell-cycle activity, cellular metabolism, and ATPase function, while the genes that increased after 1 min exposure were correlated to kinase and oxidative stress response [59]. Moreover, genes that managed acetylation and methylation were unaffected after 1 min exposure, and although several essential signaling systems, including MAPK, p53, the transforming growth factor, and the tumor necrosis factor, were stimulated by this contact, this stimulation decreased after 3 min exposure. In that study, it was also stated that the modified expression of several genes 2 h after 3 min exposure was related to variations in methyltransferases activity. Thus, 3 min CAP exposure can modify DNA methylation and provoke an increase in the genes that are relevant in histone acetylation [59].

In a different study, Lee et al. employed the H3K4me3 genome-wide ChIP-sequencing technique to assess CAP’s effect on histone methylation [60]. The results demonstrated that up to 899 sequences that fell within the promoter regions had modifications in the amount of H3K4me3 methylation in tumor cells after CAP treatment. Subsequent analysis demonstrated that the signaling systems involved were concerned with DNA repair, DNA replication, and cell cycles. It is also relevant that the expression amounts of 18 genes presented significant relationships with the degree of modified amount of H3K4me3 methylation [61].

CAP’s action on histone acetylation modifications has also been reported [62]. Whole genome RNA sequencing recognized relevant increases in the expression of 469 genes and a reduction in the expression of 941 genes after CAP treatment on adipose tissue-derived stem cells (ASC). Greater effects were reported in genes that were involved in cytokine production, while a reduced activity was described in genes that were implicated in apoptosis. Crucially, CAP-exposed cells also presented increased activity of histone deacetylase 1 (HDAC1) and reduced the levels of acetylated histone-3. The effects on HDAC1 expression were noticed 9 h after CAP treatment, but this effect was reduced after 24 h, while histone 3 acetylation reduction was at its peak 24 h after treatment. Furthermore, CAP-treated cells that were then exposed to HDAC1, DNA and histone methylation inhibitors presented lower cytokine and growth factor production [62].

A different epigenetic mechanism of the action of CAP may be secondary to its ability to act on non-coding genetic material. MicroRNAs (miRNAs) are short non-coding RNAs, 18–22 nucleotides in length, which operate as controllers of several cellular processes via a negative effect on gene expression at the post-transcriptional level. MiRNA changes have a central effect on the onset and progression of solid and hematological tumors [63,64].

CAP can change the expression and function of miRNA. For instance, changes in the activity of miR-19a-3p were described after CAP exposure in cancer cell lines [65]. This miRNA is believed to be oncomiR, which is aberrantly greatly expressed in pancreatic and gastric tumors and correlated with worse outcomes [66,67].

CAP treatment can also modify lncRNAs’ expression and function. LncRNAs are molecules that are 200 to 100,000 nucleotides long and that are not translated into protein. They are changers of the epigenetic processes and can be involved in the genesis of tumors [68]. A study showed that CAP could modify the expression of diverse genes, depending on contact times [69]. A more relevant modification in expression occurred in the *ZNRD1* gene and in its antisense lncRNA. Although continued 10 min exposure increased the expression of this gene, reiterated shorter exposure for 10 × 30 s decreased its expression. Accordingly, the CAP influence on this lncRNA expression was opposite to its effect on ZNRD1 gene expression (Figure 2).

Several studies have evaluated CAP as a possible treatment for solid malignancies. Therefore, the aim of our review was to analyze the existing works and to consider the possibility of preclinical and clinical uses of CAP in the context of hematological neoplasms. 

## 3. CAP and Hematological Malignancies

### 3.1. Acute Myeloid Leukemia

The chance of CAP-device application to leukemia cell lines has been evaluated. CAP provoked in vitro cell death in THP-1 cells (human monocytic leukemia cell line) in a dosage-dependent fashion [70]. CAP treatment caused programmed cell death 45 s after CAP exposure, while necrosis was reported after exposure for more than 50 s [71].

A further experimentation evaluated the cell death of human myeloid leukemia cells by remote contact with CAP-produced RNS by employing a resistive barrier discharge system. The results suggested that CAP exposure for 45 s caused the onset of RNS-provoked apoptosis and, for longer exposures (50 s), the development of necrotic death [36].

In addition to a direct effect on leukemic cells, CAP could operate by causing a deep alteration of gene activation. In fact, an experiment evaluated target genes that were modified by the ROS and non-ROS component of CAP [72]. After CAP or H_2_O_2_ exposure of U937 leukemia and SK-mel-147, the genetic data of melanoma cells were investigated. The findings showed 252 and 762 genes in the H_2_O_2_ -treated U937 and SK-mel-147 cells, and 112 and 843 genes in the CAP-treated cells, with expression modifications greater than double. Remarkably, only two and four genes were modified in common by CAP and H_2_O_2_, the genes were equally inhibited by both CAP and H_2_O_2_, suggesting that non-ROS components were responsible for the control of the greater part of the CAP-controlled genes. Furthermore, experiments performed employing inhibitors of ROS and nitrogen oxide synthase (NOS) showed the ROS- and reactive nitrogen species (RNS)-autonomous control of PTGER3 and HSPA6 when U937 clonal cells were exposed to CAP [72]. Accordingly, it was possible that CAP-related genes were controlled by components other than ROS or RNS.

As for the mechanism of action of CAP, a different hypothesis was formulated. Some findings suggested that CAP treatment can modify the specific metabolism of leukemic cells, thus provoking cell damage and death. Tumor cells may obtain a huge growth that is imputable to metabolic changes. Metabolic modification is a tumoral characteristic that accelerates the incorporation of carbons into molecules, such as nucleic acid, proteins, and lipids, to produce a great number of metabolites that are necessary for the expansion of tumor cells [73,74,75,76].

A report explored the metabolite profile of CAP exposure on leukemia cells evaluated on gas chromatography tandem time-of-flight mass spectrometry (GC-TOFMS) [77]. Furthermore, the authors of that report performed a bioinformatic analysis of metabolic signaling and found relevant modifications, according to basic data analysis. The findings demonstrated that glutamate, aspartate, and alanine metabolism were substantially altered after CAP exposure. The glutaminase function was reduced after CAP treatment, and this change was able to cause a glutamine amassing, which in turn was able to provoke the death of leukemia cells [77] (Table 1).

This type of approach might be advantageous in recognizing metabolic signaling that might be evaluated for CAP treatment, as the energetic condition of leukemic cells can be a specific target for killing leukemic cells more precisely.

However, the possible use of CAP in clinical conditions is uncertain, and determining the best way for its implementation requires further study.

### 3.2. Chronic Myeloid Leukemia

The use of CAP has also proved effective in controlling cell proliferation in other hematological malignancies, such as chronic myeloid leukemia (CML). Specific techniques of CAP administration have been employed in the treatment of this disease. In an experimental animal model of resistant CML, the authors considered whether trident cold atmospheric plasma (Tri-CAP), a CAP with extremely low concentration of ROS, could block different survival system pathways [78]. They showed that Tri-CAP disturbed the CML survival signaling involved in glycolysis, redox deregulation, and the AKT/mTOR/HIF-1α pathway. Furthermore, this type of CAP provoked a massive increase in programmed cell death in CML cell lines and in primary progenitor cells from CML subjects that presented with the treatment-resistant T315I mutation. Conversely, normal cells were not altered by this treatment, indicating that Tri-CAP specifically affected resistant CML cells. The analysis also showed that Tri-CAP was able to decrease disease progression in animal models, improving the survival of CML-bearing animals.

The possibility of utilizing Tri-CAP in CML patients was reported in that study. The technique might be extracorporeal for hematopoietic stem cell transplant or transdermal administration, or by its stimulated solution for infusion treatment [78]. In addition, Tri-CAP might represent a powerful approach in CML patients, especially in overwhelming chemoresistance.

As for other possible mechanisms of action, a study established that miRNAs are implicated in CAP-provoked cytotoxicity [79]. That experiment showed that 28 miRNAs were considerably modified in chronic myeloid leukemia K562 cells (11 reduced and 17 increased) after 24 h exposure with an argon CAP for 90 s with respect to unexposed cells. These miRNAs were correlated to protein binding, the cAMP signaling system, other signaling pathways such as AMPK, and phosphatidylinositol signaling [79] (Table 2).

### 3.3. Multiple Myeloma

The efficacy of CAP treatment was also demonstrated in cell lines other than myeloid. An in vitro experimentation evaluated the effects of CAP exposure on multiple myeloma (MM) cells. The authors discovered that CAP was able to cause the disconnection of adherent MM cells, and that the disconnection was related with a greater amount of hydroxyl radical in the gas phase [80].

In the same work, the authors considered whether CAP could modify MM cell differentiation by the enhanced expression of differentiation factors, such as Blimp-1 and XBP-1. CAP exposure for 2 min increased Blimp-1 and XBP-1 expression, while the expression of EBF was reduced. This result may suggest that CAP could stimulate MM cells to a more differentiated condition. The finding was confirmed by the fact that CAP exposure increased the rate of the CD138 + cells, a membrane marker of differentiation. However, the most interesting point of the study was the possibility that CAP treatment might modify the progression of the disease via the effects exerted on two fundamental elements of MM progression, such as MMP-2 and MMP-9. In fact, both elements were decreased by CAP exposure. The migration capability was also blocked by CAP via the same reduction of MMP-2 and MMP-9 secretion [80].

CAP treatment could be also useful in fighting chemoresistance, which is one of the biggest problems in MM treatment. Bortezomib (BTZ) is a proteasome inhibitor that is employed in MM treatment. Several data showed that cell survival was reduced after BTZ exposure for 24 h and 48 h. However, combined CAP exposure for 30 s or 40 s with BTZ at doses of 3 nM and 5 nM for 24 h remarkably reduced MM cell survival, with respect to either CAP exposure or BTZ administration alone. Finally, in the same work, the authors evaluated the effect of CAP on MM-programmed cell death. After CAP treatment, the MM cells presented an increased apoptosis, probably via the effect exerted on JNK, which was reduced, while eIF2a was increased [80].

The effects of CAP on MM cell apoptosis were also evaluated in another experimental study performed by the same group of researchers. CD95 is a death receptor that is essential in stimulating cancer apoptosis [81,82]. It is broadly present in patients’ myeloma cells and in MM cell lines [83,84]. A ROS increase could control CD95, therefore stimulating CD95-caused MM cell programmed death [85,86]. Xu et al. showed that He + O_2_ plasma could increase MM cell apoptosis via the stimulation of CD95 and downstream caspase cascades [87]. As stated, an ROS increase is crucial for CD95-caused apoptosis in response to CAP exposure. Of more relevance, the study established that CD95 is more significantly expressed in MM cells than in healthy cells, which suggested that CD95 could be a useful target for CAP treatment, as it could specifically destroy MM cells [87] (Table 3).

Finally, although there is no specific work on the subject, it is possible to hypothesize that CAP could represent a useful complement to the treatment of bone lesions that are present in MM patients. A recent study showed that CAP positively modifies the function of osteoblast-like cells by increasing their growth and by stimulating cell mobility and survival [88].

CAP exposure of osteoblast-like cells for 30 and 60 s caused a reduction of several factors that are involved in apoptosis, such as caspases, p53, apoptotic protease activating factor-1, BCL2 Antagonist/Killer 1, and B-Cell Lymphoma2. Moreover, a nuclear translocation of p53 and a morphological cytoskeleton alteration were reported after CAP treatment at 1 d [89]. This in vitro experiment showed that CAP could reduce programmed cell death in osteoblast-like cells, highlighting a favorable effect on hard-tissue cells. In the near future, CAP treatment-induced modification of osteoblastic activity could possibly be employed in the management of bone disease in patients with MM.

### 3.4. T-Lymphoblastic Leukemia

Within the framework of lymphoproliferative diseases, a study evaluated the proapoptotic action of CAP and its capability to modify oxidative stress in T-lymphoblastic leukemia cells (Jurkat cells) and clarified the molecular mechanism activated by CAP usage [90]. The use of CAP stimulated the production of RONS, causing the onset of cytotoxicity on tumor cells and an increased programmed cell death due to a p53-enhanced expression. The effects reverted after a longer time of treatment, probably due to a compensatory mechanism with a posttranscriptional increase of components such as CAT, superoxide dismutase 1, and glutamine synthetase R2 [90].

Programmed cell death induced by CAP treatment could be due to the mitochondrial/intrinsic pathway or the receptor/extrinsic pathway [91]. The participation of the intrinsic pathway was confirmed via a study of Bax and Bcl-2 activity [92,93], which showed increased protein expression after CAP. However, no change was identified at the RNA level. Similarly, CAP caused an increased production of Bcl-2 at the protein level, but mRNA expression was decreased 6 h after CAP treatment and increased after 24 h. The different effects on Bax and Bcl-2 at the protein and mRNA levels could be secondary to the mechanisms implicated in the posttranscriptional control to change mRNA into protein [94]. Furthermore, the reported increase in Bcl-2 protein may operate as a compensatory defense of Jurkat cells after CAP exposure [95]. In fact, it has been reported that cells that present an increased amount of Bcl-2 showed a sub-pathological increase in ROS production that promoted the antioxidant defense. Finally, the various effects of CAP on Bcl-2 mRNA might be due to other compensatory systems, such as epigenetic mechanisms [96].

## 4. CAP and Stem Cell

The effects of CAP could be exerted not only on cancer cells but also on cancer stem cells (CSCs) themselves. In fact, CAP may also operate specifically on CSCs, as ROS generation generally coincides with GSH extrusion [97]. The great amount of GSH extrusion is one characteristic of all less-differentiated cells, including CSCs [98]. This is because CSCs have a rather small redox amount, due to their greater antioxidant ability, and CAP can regulate CSCs’ activity by concurrently decreasing their antioxidant capabilities while fostering redox variations.

However, CAP seems to present completely different effects in normal stem cells. This finding might be conveniently evaluated for the cure of several diseases, such as bone marrow aplasia or the reestablishment of hematopoietic normality after a bone marrow transplant.

CAP is an exceptional approach to modify the fate of stem cells. It can regulate cell fate by operating on the cell-resident niche, and it can change cell fate by activating stem cells in close contact, as it is able to provoke chemical modifications on the cells’ culture surfaces. One of the crucial factors of surface chemistry is surface energy, which is generally reflected by wettability. Ueda et al. ascertained that pluripotent stem cells (PSCs) adhered inadequately to hydrophobic cell culture dishes [99], but after CAP treatment of the hydrophilic surface, the chemically changed polystyrene face could sustain ideal PSC attachment and long-lasting self-renewal. However, a novel hypothesis was formulated, proposing that CAP treatment is able to change other surface characteristics [100]. CAP exposure of the polymeric substratum also modified the elasticity at the nanoscale size. The main mechanical effect enhanced the adhesion of human mesenchymal stem cells (MSCs) via efficacious focal merging. The persistent self-renewal and growth due to CAP could pave the way for the use of this technique in regenerative medicine.

CAP treatment seems to be capable of increasing the proliferation of normal stem cells, while preserving their stemness. The effects on cell growth are obtained via an indirect activation of the cells that are present in the niche and by a direct effect on stem cells.

A study reported enhanced growth of unrestricted somatic stem cells (USSCs) on a polystyrene surface treated with argon CAP or oxygen CAP, compared with untreated samples [101].

In another study, the growth of BM-MSCs was remarkably greater on the CAP-treated gelatin films than on the untreated ones [102]. Tan et al. confirmed these findings and clarified their mechanisms [103]. It was proposed that the cell growth on plasma-treated surfaces was the consequence of quicker advancement of the cell cycle, probably due to a privileged production of focal adhesion kinase.

The CAP effects on stem cells were confirmed by different experiments [62,104,105]. In 2016, a study concluded that helium-based CAP increased the proliferation of adipose tissue-originated stem cells by nearly 60% after 3 days of incubation, compared with the proliferation of untreated cells [104].

Furthermore, CAP can stimulate the lineage-specific differentiation of stem cells into different tissues, such as teeth, bone, or cartilage [106,107,108,109].

The use of CAP can also stimulate growth in bone marrow-originated stem cells, doubling the growth rate of untreated cells [94]. In treated cells, an increase in the expression of *OCT4*, *SOX2*, and *NANOG* genes was reported, and the expression of genes that operate on G1-S cell-cycle transition also increased, suggesting that the use of CAP can regulate this cell cycle phase [105].

## 5. Future Perspectives

The antitumor effects of CAP helped to establish a new discipline in medical research called “plasma oncology” [110]. As a developing discipline, the medical usage of CAP as a possible tumor treatment is at very early phases of investigation. In fact, the first case where CAP was employed as a cancer treatment was only approved by the FDA in 2019, for a young man who had a relapsed incurable peritoneal sarcoma and for another person who had a late-stage pancreatic tumor [111].

Further studies are needed to confirm the therapeutic efficacy of CAP against hematological malignancies. In fact, most of the works reported in our review were conducted in vitro or in vivo using experimental animal models. Currently, only three clinical trials are registered for the treatment of cancer or precancerous skin lesions (www.clinicaltrial.gov (accessed on 10 June 2022)), while another seven clinical trials are registered for the treatment of skin wounds or skin infections (Table 4). Furthermore, clinical trials are generally conducted at highly specialized centers, making the implementation of large studies more difficult. However, numerous spontaneous studies have been conducted on small numbers of patients.

In the near future, several aspects of CAP treatment will need to be investigated, numerous doubts resolved, and numerous methodological and technical problems overcome before this technique can be translated into clinical practice.

The indirect interaction between CAP and cells means that CAP, a slightly used method, will be increasingly used in the future, with fewer collateral effects than those of traditional chemotherapy. Nevertheless, CAP-generated ROS can also enter normal cells, and whether and how this increase might provoke a changed signal transduction in the tumor milieu that influences tumor state transitions has not been entirely studied and requires further analysis [22,112,113].

Moreover, due to the presence of multiple components in CAP, each of which causes different biological effects in several cells, it is impossible to determine general CAP-treatment approaches. In addition, the clarification of the fundamental molecular processes via which each CAP constituent blocks clonal cell proliferation is essential in instituting tumor treatment schemes. For instance, as the amount of RONS and the dosage schedule are the two most relevant factors in determining the effectiveness of the treatment, a precise measurement of the RONS during in vivo experiments is pivotal. Uniform methodologies are essential in guaranteeing, for example, the same interval between tumor onset and the start of CAP treatment.

It should also be borne in mind that different forms of clonal cells responded to CAP with different modalities, as confirmed by the remarkably diverse groups of modified genes, with approximately 50% of the altered genes displaying different profiles in terms of CpG methylation [114]. Furthermore, a different modality of exposure may justify a different CAP effect. A study reported that CAP treatment for 600 s had opposite controlling effects than undergoing 10 fractions of 60 s each on the ZNRD1 gene [69], which is involved in drug resistance to leukemia [115,116]. A different experiment showed the generation of a proapoptotic or a proliferative effect on tumor cells depending on CAP-treatment situations [117]. In addition, slight variations in the experimental system, including the CAP device or the chemical configuration of the culture medium, might influence the results.

Conversely, a study that elaborated and took into account different treatment conditions, such as multiple H_2_O_2_ amounts and diverse CAP exposure conditions, was useful in reaching a complete regulatory pattern [118,119,120].

In any case, CAP treatment may have several advantages in subjects with hematological malignancies and could be useful either alone or in combination with traditional therapeutical approaches. In fact, CAP reestablishes cell responsivity to chemotherapy treatment [121]. This has been ascribed to an extremely active combination of activated and non-activated species, and to a possible synergistic action arising from the interaction between these species and CAP-produced physical consequences targeting diverse pathways, including the cellular antioxidant system [122].

Furthermore, CAP has the possibility to enhance the efficacy of traditional or innovative drugs. It can be employed, with synergistic results, with nanoparticles, enzymes, and other therapeutical systems, such as photodynamic therapy. The synergistic effect of CAP application might be useful in decreasing dosage quantities of chemotherapeutics, while maintaining effectiveness. Finally, CAP usage might, in some cases, replace radiation treatment, which has noxious collateral effects.

The combined administration of 1 µM cisplatin with 3 min CAP exposure or 3 µM cisplatin and 1 min CAP treatment was employed to obtain a synergistic result on SCC-15 cells. In fact, the utilization of 1 µM of cisplatin without CAP provoked a reduced survival in only 25% of SCC-15 cells, while its combined use with 3 min CAP exposure provoked a 60% reduction in cell survival. Although cisplatin has been employed as a treatment in several hematological and solid tumors, its administration provokes several serious and unwanted collateral effects [123]. Therefore, CAP usage may cause an increased cisplatin effectiveness, but allow for decreased dosages. As for the mechanism, the combined administration of CAP and cisplatin provoked a more marked expression of some genes that are implicated in programmed cell death, such as PTEN, caspase 9, and p53, compared with the expression that was obtained by either cisplatin or CAP application alone. Overall, apoptosis was more marked in cancer cells than in fibroblasts [124].

Similarly, the toxic action of doxorubicin or epirubicin was obtained at a sub-micromolar dose, with a 10-fold lower amount than the classical Ic50. Furthermore, there was no increased micro-nuclei generation in CAP-only treatment, and CAP fostered SLC22A16 cationic transporter concentrations at the molecular level; this compound was reported as a doxorubicin importer [125].

Treatment with CAP or dacarbazine (DAC) under in vivo situations caused mass reduction of tumors in an animal experimental model, but the most relevant tumor decrease was reported when a combined treatment was employed [126].

CAP capacity is not restricted to a synergistic effect with traditional therapeutical drugs, as it also increases the efficacy of nanoparticle treatments that are presently being utilized to decrease the side effects of chemotherapeutics [127,128,129,130,131].

It is possible that CAP treatment provoked membrane injury, which stimulated a membrane repair response with the onset of a quick endocytosis that was able to eliminate damaged elements and increase membrane permeability. This effect provoked an enhanced cell Au nanoparticle absorption, and successively increased cytotoxicity produced by nanoparticles [132].

However, different experimental conditions may provoke different effects. Platinum nanoparticles (Pt-NPs) are powerful antioxidants with a relevant capacity to scavenge reactive species. The effects of Pt-NPs on He-CAP-caused programmed cell death were evaluated in the lymphoma U937 cell line. Remarkably, Pt-NPs scavenge He-CAP- stimulated reactive species and block all the pathways that are implicated in programmed cell death dynamics. This might be due to the SOD/catalase mimetic actions of Pt-NPs [133].

One of the most likely future applications of CAP is its use in tumor immunotherapy, as its immune system’s modulation capability overwhelms tumor cells’ attitude to inhibit immune reactions [134,135,136]. Cells altered by chemotherapy induce the formation of “damage-associated molecular pattern signals” (DAMP), which stimulate the immune system to kill these cells. Remarkably, some experimental studies proposed that CAP stimulated immunogenic cell death (ICD), and that this provoked macrophage stimulation [137,138]. Cheng et al. reported that 30 s of CAP stimulation of macrophages caused an increased generation of cytokines, such as IL-2, IL-6, IL12, and IFN gamma, and a reduction of IL-10 [139].

Immune checkpoint blockade (ICB) treatment increases antitumor response by blocking immune suppressor factors, including programmed cell-death protein 1 and its ligand (PD-1/PD-L1) and cytotoxic T-lymphocyte-associated antigen 4 (CTLA-4) [140].

An experiment evaluated the effect of a combined use of CAP and ICB therapy based on a hollow-structured microneedles (MN) platform [141]. Hollow-structured microneedles (hMNs) were prepared to ease the transdermal diffusion of CAP to the tumor cells and stimulate the immunogenic death of tumor cells. Moreover, the discharged tumor-correlated antigens could operate as “danger signals” to stimulate the maturation of dendritic cells, after a stimulated T cell-mediated antitumor response. The CAP-induced immune response was increased by the aPD-L1 that was loaded inside the MNs. In contrast, only CAP and CAP/solid-structured MNs did not modify tumor growth, most likely due to the small penetration of CAP. The presence of CD4+ and CD8+ T cells significantly increased after the combined administration of CAP and aPD-L1–hMNs [141]. These findings indicated that the microneedle design allowed an increase in the efficacy of an ICB inhibitor by combined use with different treatments, such as transdermal CAP treatment.

However, in the future, it will be necessary to evaluate the possibility of increasing the effectiveness of the technique. In a study, electron paramagnetic resonance (EPR) spin trapping and flow cytometry were employed to recognize the free radicals produced by employing argon-cold atmospheric plasma (Ar-CAP) in aqueous solutions and intracellularly, in comparison with those produced by X-irradiation [142]. Human lymphoma U937 cells were employed to analyze intracellular oxidative stress. The study demonstrated the generation of huge quantities of OH radicals employing Ar-CAP, compared with that generated by X-irradiation. Small amounts of H atoms were highlighted, while nitric oxide and pyrolysis were not identified. In spite of the superiority of Ar-CAP in generating OH radicals, the exposure to X-rays turned out to be more lethal.

## 6. Conclusions

In the near future, the use of the CAP may find new fields, definitively expanding on its subordinate role as a mere surgical tool in cancer treatment, to which it has often been relegated up.

However, the use of CAP in hematological diseases will face specific challenges (Table 5). 

It will be necessary to move from the simple direct damaging action exerted on the neoplastic cells, as in the case of skin tumors, to more complex therapeutic strategies. These treatment methods must use all the therapeutic possibilities of the technique and, depending on the hematological pathology, enhance the effect induced by oxidative stress, taking advantage of the possibility of inducing an immune-mediated toxicity or stimulating a new sensitization to the cytotoxicity induced by chemotherapeutics.

Further studies carried out on patients will allow us to better define the possible side effects induced by CAP therapy, although the studies conducted so far have not highlighted the appearance of serious collateral effects. A clinical safety assessment of CAP has been conducted ex vivo on human skin, where a treatment of up to 2 min has been shown to safely avoid DNA damage [143]. Moreover, there was no significant adverse effect reported in a study of seven patients with skin erosion wounds after CAP treatment. The CAP jet seemed able to cause a mild sting only during the therapy, and all subjects stated that it could be tolerated without discontinuing the treatment [144].

In a different experimental animal study, CAP did not provoke relevant changes in red cell parameters, such as red blood cell count, average of corpuscular volume, corpuscular hemoglobin concentration, and corpuscular hemoglobin, indicating that CAP did not produce collateral effects on animal red blood cells. Similarly, the liver functionality assay did not demonstrate relevant modifications among animals after CAP treatment, further demonstrating the safety of physical plasma as an onco-therapy [109]. However, the follow-up after the studies was too short to rule out long-term adverse effects.

Similarly, a wider use of the CAP would perhaps make it possible to reduce costs that are necessary for the implementation of the technique; however, such costs would not be excessive. In this sense, the widespread use of the CAP for such uses as microorganism sterilization, biofilm inactivation, and wound healing has made it possible to reduce the costs of the procedure.

In any case, the costs of the treatment are closely linked to the type of device that is used. For instance, the cost of InvivoPen is lower than the cost of the plasma-activated medium, as it does not need liquid as the media to confer CAP efficacy [119].

In conclusion, it cannot be ignored that the use of CAP use has some minimal negative effects at the molecular level. All the data and possibilities are subject to ongoing analysis, but the current results suggest that the unproven adverse effects are outweighed by CAP’s many benefits [145,146].

## Figures and Tables

**Table 1:** Effect and mechanisms of action of CAP on acute leukemic myeloid cells.

Cells	Effect and Mechanism	Type of Study	Refs.
THP-1 cells	Apoptosis and necrosis.	In vitro	[70,71]
Human myeloid leukemia cells	Apoptosis and necrosis.	In vitro	[36]
U937 cells	Gene expression.	In vitro	[72]
U937 cells	Metabolic changes (glutamine amassing).	In vitro	[77]

**Table 2:** Effect and mechanisms of action of CAP on CML cells.

Experimental Model	Effect and Mechanism	Type of Study	Ref.
Animal model of resistant CML	Apoptosis. Altered glycolysis and redox deregulation. Alteration of AKT/mTOR/HIF-1alpha pathways.	In vivo	[78]
CML cell lines. Primary progenitor CML cells with T315T mutation	Apoptosis.	In vitro	[79]
K 562 cells	MirNAs alteration (protein binding, AMPc signaling).	In vitro	[79]

**Table 3:** In vitro study on CAP effects on multiple myeloma cells.

Type of Cells	Effect and Mechanism	Type of Study	Ref.
MM cell lines	Modification of differentiation factors (BLIMP-1 and XBP-1). Reduction MMP-2 and MMP-9 Effect on bortezomib chemoresistance. Apoptosis (reduction of JNK, increased eIF2a).	In vitro	[80]
MM cell lines	Apoptosis (stimulation of CD95, caspase activation	In vitro	[87]

**Table 4:** Clinical trials registered for the treatment of neoplastic diseases and precancerous lesions via the use of CAP (www.clinicaltrial.gov (accessed on 10 June 2022)).

NCT Number	Study Title	Conditions	Interventions
NCT03218436	Physical Cold Atmospheric Plasma for the Treatment of Cervical Intraepithelial Neoplasia	Cervical intraepithelial neoplasia	Treatment with low-temperature argon plasma during colposcopic examination.
NCT02759900	Using a Cold Atmospheric Plasma Device to Treat Skin Disorders	Skin lesions, precancerous conditions	Non-thermal, atmospheric plasma treatment of affected area or lesions using a nanosecond dielectric barrier discharge plasma device.
NCT05070754	Cold Atmospheric Plasma Device for Pediatric Molluscum and Verruca	Verruca vulgaris Molluscum contagiosum	Floating electrode-dielectric barrier device (FE-DBD) cold atmospheric plasma.

**Table 5:** Aspects of current status and prospects of plasma oncology.

Advantages of Cap Treatment	Less potential adverse effects, compared with traditional chemotherapy Possible synergistic action with traditional chemotherapy or immunotherapy Possibility of creating new experimental models for the study of hematological neoplasms
Disadvantages of Cap Treatment	Need for specialized centers provided with appropriate equipment Special training of the personnel required Difficulty in assessing the therapeutic effects of the treatment a priori
Open Questions	Effects on bone marrow microenvironment Need to evaluate long-term side effects Need to evaluate the best treatment modalities, with regard to timing and doses, to obtain the best synergistic effects with traditional chemotherapy or immunotherapy Need for large randomized clinical trials to evaluate the real efficacy of CAP treatment
